# Phenolics in* Primula veris* L. and* P. elatior* (L.) Hill Raw Materials

**DOI:** 10.1155/2017/2871579

**Published:** 2017-08-01

**Authors:** Katarzyna Bączek, Jarosław L. Przybył, Małgorzata Mirgos, Olga Kosakowska, Izabela Szymborska-Sandhu, Zenon Węglarz

**Affiliations:** Laboratory of New Herbal Products, Department of Vegetable and Medicinal Plants, Warsaw University of Life Sciences-SGGW, Nowoursynowska 166, 02-787 Warsaw, Poland

## Abstract

*Primula veris* L. and* Primula elatior* (L.) Hill represent medicinal plants used for the production of herbal teas and preparations with antioxidant and expectorant activity. Flowers and roots of both species possess the same biological activity. In the presented study, raw materials of wild growing* P. veris* and* P. elatior *were compared in terms of the content and composition of phenolic compounds using a fast and simple HPLC-DAD method. The study showed that flowers of both species were rich in flavonoids. However,* P. veris* flowers were characterized with a distinctly higher content of isorhamnetin-3-O-glucoside, astragalin, and (+)-catechin, whereas* P. elatior* occurred to be a richer source of rutoside and isorhamnetin-3-O-rutinoside. Hyperoside was found exclusively in* P. elatior* flowers. Phenolic glycosides (primverin and primulaverin) were identified only in the roots. Their content was about ten times higher in* P. veris* in comparison with* P. elatior* underground organs. The obtained results clearly show that both* Primula* species differ distinctly in terms of the content and composition of phenolic compounds. The compounds differentiating both species to the highest degree (hyperoside, in flowers, as well as primverin and primulaverin, in the roots) may be useful chemical markers in the identification and evaluation of both species.

## 1. Introduction

Cowslip (*Primula veris* L., syn.* P. officinalis* Hill) and oxlip (*Primula elatior* (L.) Hill) are small, long-lived perennials from the family Primulaceae, growing wild in temperate Europe and Asia [1]. Cowslip grows on nutrient-poor grasslands, herb-rich meadows, and at the edges and in clearings of warm and bright woodlands. Oxlip prefers moist and shaded forests, but it also grows in mountain meadows [2, 3]. Both species produce a rosette of leaves and leafless flower stalks, up to 20–30 cm high. Cowslip flowers are fragrant, bright-yellow with orange spots at the edge of each lobe. They are formed at the top of the stalks in an umbel-like inflorescence. In turn, the pale-yellow, almost scentless, flowers of oxlip are produced on separate stalks. In the central part of these flowers an orange ring is visible [1, 2]. Underground organs consist of slightly curved, grayish-brown rhizomes with yellowish-white* (P. veris)* or brown* (P. elatior)* roots, commonly called roots [1, 4].

Both species have a long history of medicinal use. In the current (fifth) edition of the European Pharmacopeia, they are listed as a source of* Primula* roots [4]. However, in the British Herbal Pharmacopeia [5] as well as in Pharmacopee Française [6], only* P. veris* is mentioned as a source of* Primula* raw materials.

*Primula veris* and* P. elatior* have mainly been exploited for the production of herbal teas and preparations that are also considered dietary supplements [1]. They indicate various pharmacological activities, for example, secretolytic, expectorant, anti-inflammatory, diuretic, antimicrobial, antifungal, and sedative [7–10]. According to EMA,* Primula* flowers and roots are used against coughs, bronchitis, and catarrhs of the respiratory tract and also to treat nervousness, headache, or rheumatism [7, 8]. In the past,* Primula* leaves and flowers were also eaten raw or cooked as a source of vitamins and microelements available in late winter [11]. Apart from* P. veris* and* P. elatior*, other* Primula* species are described as also revealing some medicinal potential. According to Demir et al. [12],* P. vulgaris* demonstrates antioxidant activity. Extracts from* P. denticulata* show cytostatic properties, while* P. macrophylla* shows antifungal ones [13–15].

The main active compounds of* Primula* flowers and roots are triterpene saponins as well as phenolic compounds, including flavonoids (about 3% in flowers), phenolic acids, and phenolic glycosides [7, 8]. Saponins are responsible for secretolytic and expectorant activity. In turn, phenolic compounds, present especially in* Primula* flowers, reveal antioxidant, antimicrobial, and cytostatic properties [12, 13].

Phenolic compounds can be easily separated on a C18 reversed-phase (RP) column and detected using a UV or diode array detector (DAD) [16–19]. All these substances contain at least one aromatic ring and thus efficiently absorb UV light. So, the UV spectra obtained by the DAD are a valuable indicator in screening and preliminary qualitative analyses of the different groups of phenolics. For better structure elucidation of metabolites and/or unambiguous identification of target compounds, liquid chromatography mass spectrometry (LC-MS) techniques or even nuclear magnetic resonance (NMR) detection are used [17–19].

Based on European Medicines Agency (EMA) and European Pharmacopeia monographs,* Primula* preparations are produced exclusively out of* P. veris* and* P. elatior* raw materials, which are considered to possess the same values [4, 7, 8]. Due to the developmental and morphological similarities between both species, they are hard to differentiate on natural sites, and after the drying process the raw materials collected from them are indistinguishable. Despite the above regulations, some authors report differences between these two species in terms of their chemical composition [1, 7, 8]. Other herbal raw materials, even those described in pharmacopeias, are very often provided by two or even three plant species. For example, the lime flower is collected from* Tilia platyphyllos* Scop.,* Tilia cordata* Mill, and their hybrid* Tilia* ×* vulgaris* [4]. Numerous studies confirm that the quality of such materials may be highly diversified, which is undesirable from an industrial point of view [20–22].

The aim of our study was to compare wild growing* P. veris* and* P. elatior *in terms of the accumulation of phenolic compounds as chemical markers for species identification and more accurate assessment of raw materials, in the context of their potential usage, by a simple, but reliable, analytical method on a standard HPLC-DAD system.

## 2. Materials and Methods

### 2.1. Plant Material

**Figure 1:**
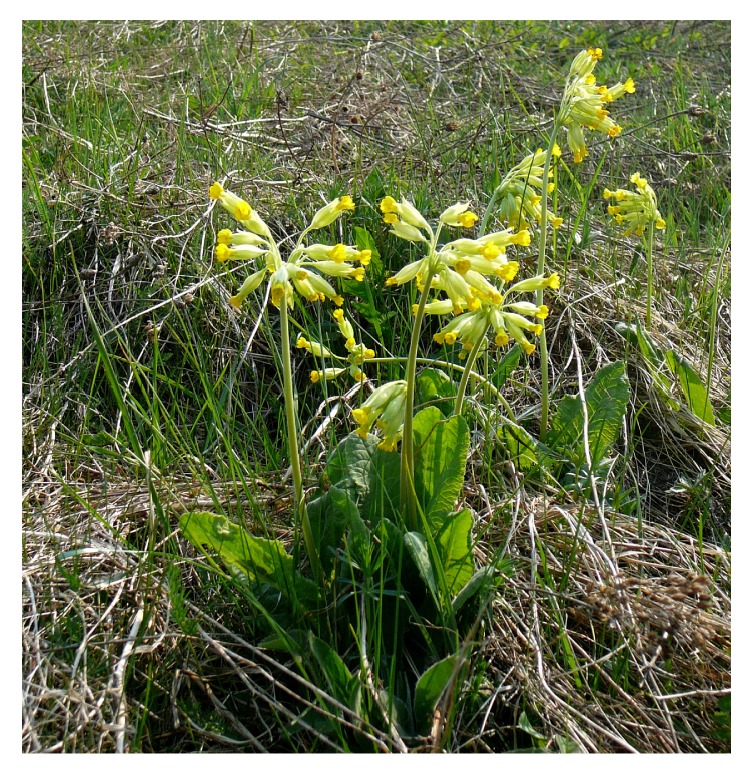
Cowslip* (Primula veris)*.

**Figure 2:**
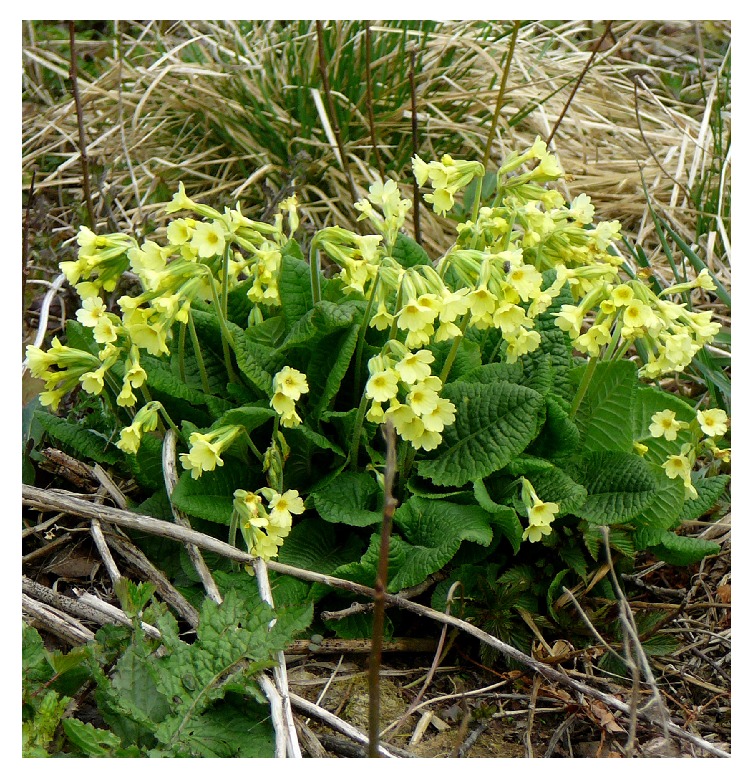
Oxlip* (Primula elatior)*.

Flowers and roots of* P. veris* and* P. elatior* were collected in the eastern part of Poland from eight wild growing populations of each species. The plant material from one population was used as one replication for chemical evaluation. Flowers were collected at the stage of full flowering (in May, Figures 1 and 2) from randomly chosen plants (about 150 g of fresh flowers per population). Roots were harvested from the same populations in September, after seed setting (about 500 g of fresh roots per population). They were washed and cut into pieces. Both sets of raw materials were dried at 40°C. Voucher specimens were taken from each population. These are stored in the Department of Vegetable and Medicinal Plants, WULS-SGGW.

### 2.2. HPLC Analysis

Air-dry, finely powdered, and homogenized raw material (1.000 g) was extracted with 100 ml of methanol (Sigma-Aldrich, Poznań, Poland, reagent grade) in a Büchi Labortechnik AG B-811 Extraction System. Soxhlet hot extraction was used with twenty-five extraction cycles, flushing and drying. After evaporation of solvents, the residue was dissolved in 10 ml of methanol. The obtained extracts were filtered with a Supelco Iso-Disk™ Syringe Tip Filter Unit, a PTFE membrane, diameter 25 mm, pore size 0.20 *μ*m and injected in triplicate. Separation was achieved using a modern C18 reversed-phase, Kinetex™ 2.6 *μ*m, 100 mm × 4.60 mm column with a porous outer layer on solid silica core particles (Phenomenex®, USA). The analyses were performed on a Shimadzu chromatograph equipped with an SIL-20A autosampler, an SPD-M10A VP PDA photodiode array detector, and CLASS VP™ 7.3 chromatography software (Shimadzu, Kyoto, Japan). The content of the determined compounds was calculated in mg per 100 g of dry weight (DW).

The analysis of flower extracts was carried out using a binary gradient of deionized water adjusted to pH 3 with phosphoric acid (Sigma-Aldrich, Poznań, Poland, reagent grade) (mobile phase A) and ACN (Sigma-Aldrich, Poznań, Poland, gradient grade) (mobile phase B) as follows: 0.01 min, 12.5% B; 4.0 min, 23% B; 6.0 min, 60% B; 6.1 min, 12.5% B; 10 min, stop. The flow rate was 1.5 ml/min, oven temperature 40°C and injection volume 1 *μ*l. Data were recorded at wavelength of 206 nm for (+)-catechin, 254 nm for luteolin 8-C-glucoside (orientin), quercetin 3-O-rutinoside (rutoside), quercetin 3-O-galactoside (hyperoside), isorhamnetin-3-O-rutinoside, isorhamnetin-3-O-glucoside, 264 nm for kaempferol 3-O-glucoside (astragalin), and 330 nm for 3-O-caffeoylquinic acid (chlorogenic acid).

For separation of root extract compounds, a binary gradient of deionized water adjusted to pH 3 with phosphoric acid (Sigma-Aldrich, Poznań, Poland, reagent grade) (mobile phase A) and ACN (Sigma-Aldrich, Poznań, Poland, gradient grade) (mobile phase B) was used as follows: 0.01 min, 18% B; 2.50 min, 20% B; 2.51 min, 95% B; 3.50 min, 95% B; 3.54 min, 18% B, 5 min, stop. The flow rate was 1.3 ml/min, oven temperature 32°C, and injection volume 1 *μ*l. Compounds were monitored at wavelength of 254 nm for primverin and 300 nm for primulaverin.

Peak identification was confirmed by comparison of retention time and UV spectra with adequate parameters of standards. Commercially available standards of the investigated compounds (ChromaDex®, Irvine, USA) were separately dissolved with methanol (Sigma-Aldrich, Poznań, Poland) in a 10 ml volumetric flask according to the ChromaDex's Tech Tip 0003: Reference Standard Recovery and Dilution and then used as standard stock solutions (https://www.chromadex.com/media/2126/techtip0003-recoverydilutionprocedures_nl_pw.pdf). Working standard solutions were prepared by dilution of 10, 50, 100, 200, 500, or 1000 *μ*l stock solutions of each compound with methanol in 10 ml volumetric flasks. The working solutions were injected (10.0 *μ*l) on a column in six replicates (*n* = 6) using SIL-20A autosampler (Shimadzu, Kyoto, Japan) to generate a six-point calibration curve. Standard curve parameters were calculated using Microsoft Excel 14 (Table 1). The signal-to-noise (S/N) ratio approach was used to determine LOD (S/N of 3 : 1) and LOQ (S/N of 10 : 1).

### 2.3. Statistical Analysis

Data were subjected to statistical analysis using Statgraphics Plus for Windows v. 4.1 software. The mean values were compared using one-way analysis of variance (ANOVA) and expressed as means with standard deviation (SD) and coefficients of variation (CV%). The differences between individual means were considered to be significant at *p* < 0.01.

## 3. Results and Discussion

According to Wichtl [1], the total content of flavonoids in* Primula* flowers is up to about 3%. To date in* P. veris *extracts, quercetin, quercetin-3-O-rutinoside, quercetin-3-O-gentiobioside, quercetin-trihexoside, kaempferol, kaempferol-3-O-diglucoside-7-O-glucoside, kaempferol-3-rutinoside, kaempferol-3-O-galactoside-rhamnoside-7-O-rhamnoside, luteolin, isorhamnetin, isorhamnetin-3-O-glucoside, isorhamnetin-3-O-rutinoside, limocitrin-3-O-glucoside, limocitrin-3-O-rutinoside, apigenin, catechin, epicatechin, and epigallocatechin, as well as some methoxylated flavones, have been identified using LC-MS and HPLC techniques [16–18, 23, 24]. Data on the composition of* P. elatior *flowers are much more scarce. These indicate the presence of rutoside, kaempferol-3-rutinoside, and isorhamnetin-3-glucoside [1].

**Figure 3:**
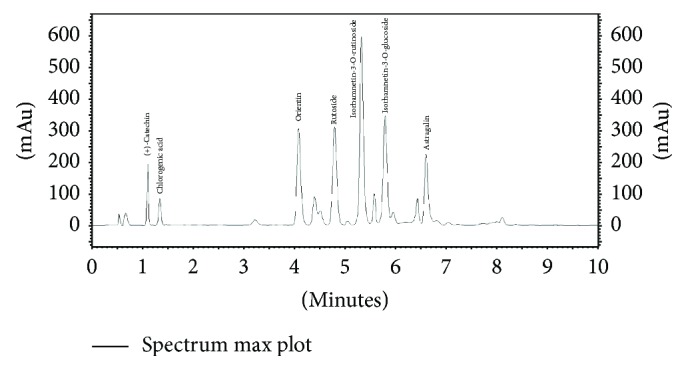
HPLC chromatogram of methanolic extract of the flowers of* Primula veris*.

**Figure 4:**
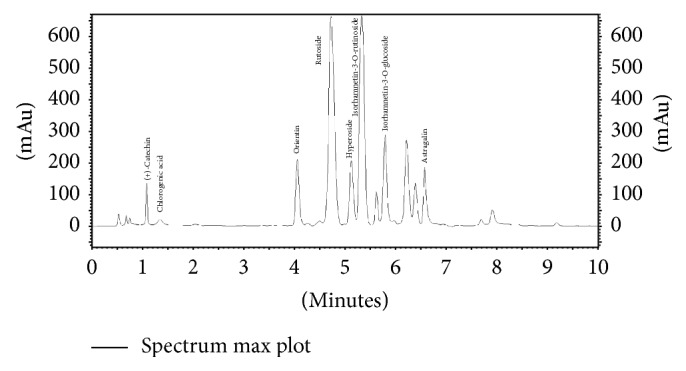
HPLC chromatogram of methanolic extract of the flowers of* Primula elatior*.

The above authors report only the chemical composition of flavonoids isolated from both* Primula* flowers. There is little information on the content of those substances. According to Wichtl [1], flowers of* P. elatior* are characterized by a higher content of rutoside (0.54%) than the flowers of* P. veris* (0.16%). Our results confirm the presence of six flavonoid compounds in the flowers of both species, namely, orientin (luteolin-8-C-glucoside), rutoside (quercetin 3-O-rutinoside), isorhamnetin-3-O-rutinoside, isorhamnetin-3-O-glucoside, astragalin (kaempferol-3-O-glucoside), and (+)-catechin. The contents of isorhamnetin-3-O-glucoside, astragalin, and (+)-catechin were distinctly higher in the flowers of* P. veris*, that is, 448.45, 185.07, and 312.11 mg/100 g DW, respectively. In turn, rutoside and isorhamnetin-3-O-rutinoside were detected in higher amounts in* P. elatior *(1025.96 and 1142.72 mg/100 g DW, resp.). A clear difference between both species concerned the presence of hyperoside (quercetin 3-O-galactoside), which was only identified in* P. elatior* flowers (265.30 mg/100 g DW) (Table 2, Figures 3 and 4). Among the analyzed substances, the content of this compound was also the most diversified (CV 46.26%).

According to Kim et al. [25], hyperoside indicates anti-inflammatory and antioxidant activities. Results obtained by Wu et al. [26] show that this compound reveals antiviral activity, while Kohlműnzer [27] also mentions diuretic and hypotensive effects. In turn, rutoside is known for its strong antioxidant potential as well as antimicrobial and anti-inflammatory activities [28]. Thus, this may explain the application of flowers of both* Primula* species in the treatment of coughs and respiratory tract diseases. The results of this study show that both hyperoside and rutoside differentiated the investigated species to a considerable degree. Therefore, flowers of* P. elatior*, which are rich in hyperoside and characterized by a higher content of rutoside in comparison to* P. veris*, may indicate stronger pharmacological activity. Unlike in the data presented by Wichtl [1], both species contained isorhamnetin-3-glucoside in their flowers (Table 2, Figures 3 and 4). Similar to hyperoside, the diversity of the content of both isorhamnetin derivatives was very high. However, the content of isorhamnetin-3-O-rutinoside was diversified to a higher degree for* P. veris* (CV 45.54%), while for isorhamnetin-3-O-glucoside this was seen in* P. elatior* (CV 43.31%). According to Teng et al. [29], isorhamnetin aglycon reveals cytotoxic activity toward human hepatocellular carcinoma cells. In our study, the presence of one phenolic acid (chlorogenic acid) in both* Primula* flowers was also confirmed, and its content was similar in* P. veris* and* P. elatior *(72.84 and 55.38 mg/100 g DW, resp.).

**Figure 5:**
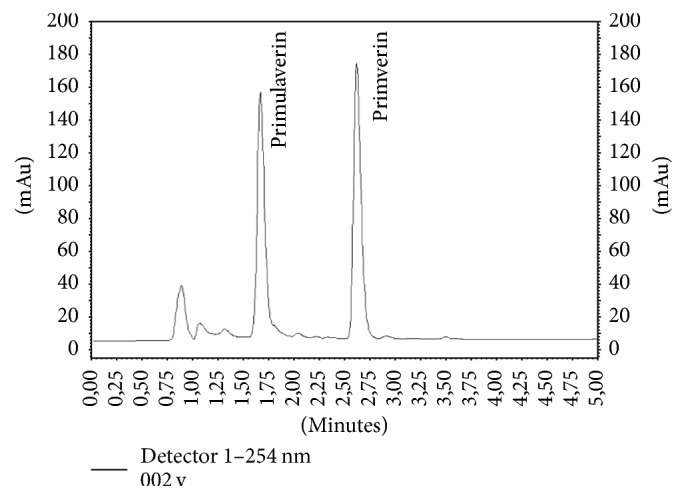
HPLC chromatogram of methanolic extract of the roots of* Primula veris*.

**Figure 6:**
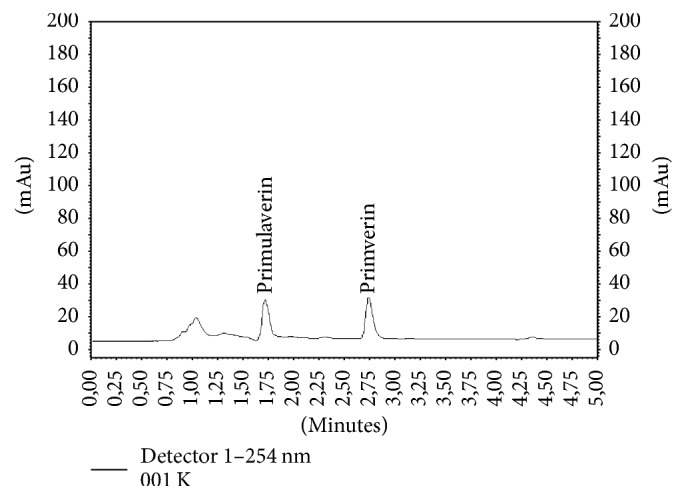
HPLC chromatogram of methanolic extract of the roots of* Primula elatior*.

Primverin and primulaverin (phenolic glycosides) are typical compounds of* P. veris* and* P. elatior* underground organs. The presence of these substances in* Primula* roots had been previously confirmed by Müller et al. [18]. According to EMA [8], their content in both species is very diversified and may be as high as 2.3%. They are responsible for the specific odor of the raw material, which appears during the drying process [1]. In our study, the content of both compounds was ten times higher in* P. veris* (1183.32 and 536.16 mg/100 g DW, resp.) than in* P. elatior* (110.31 and 74.40 mg/100 g DW, resp.) roots (Table 3, Figures 5 and 6). Such a relationship had previously been reported only for primverin [18]. In addition, the results of our study confirm that the content of primverin was much less diversified in both species than the content of primulaverin (Table 3).

According to our results, the use of a column with porous outer layer on solid silica core particles significantly reduces the analysis time and mobile phase consumption in comparison to existing methods [17, 18]. As a result, the analysis of phenolics in Primula raw materials can be effected faster and at lower cost and can be performed on standard (older) chromatographic systems.

The data concerning the chemical profile of other* Primula* species are fragmentary. Hashimoto et al. [30] identified three flavonol glycosides in* P. sieboldii *flowers and leaves, that is, quercetin and kaempferol derivatives, as well as two anthocyanins, that is, malvidin and petunidin glycosides, which were detected only in the flowers. In turn, Ozkan et al. [31] used HPLC to assess the content of catechin, rutin, and some phenolic acids, namely, gallic, protocatechuic, p-OH benzoic, vanillic, and p-coumaric acids in* P. vulgaris* flowers. According to this analysis, rutin and p-coumaric acid seemed to be the main phenolic compound of this raw material. In other* Primula* species, that is,* P. denticulata*,* P. auricular*,* P. halleri*,* P. malacoides*, and* P. marginata*, primetin (5,8-dihydroxyflavone), which is responsible for strong sensitizing properties, was also detected [32]. Depending on the chemical composition and content of biologically active compounds, different plant species of the genus have been used for various medicinal purposes, such as food poisoning, indigestion, dysentery, and ulcers as well as coughs or bronchitis, which are typical ailments treated with* P. veris* and* P. elatior* extracts.

## 4. Conclusions

Our results show distinct differences in terms of the content and composition of phenolic compounds identified in* P. veris *and* P. elatior* raw materials.* Primula elatior* flowers seem to be an interesting source of flavonoids. They are rich in rutoside and hyperoside, which reveal numerous pharmacological activities, that is, anti-inflammatory, antioxidant, and antimicrobial. Thus, they can be considered more interesting for the herbal medicine industry than* P. veris*. In turn, hyperoside was only found in the flowers of* P. elatior*, which may be used in the identification of* Primula* species. Flowers of neither species contained primverin or primulaverin. These substances were only identified in the roots.* Primula veris* was characterized by a ten times higher content of both phenolic glycosides in comparison with* P. elatior*.

Phenolic compounds identified in our study, especially hyperoside, primverin, and primulaverin, may be applied as chemical markers in the identification of* Primula* species as well as quality markers for their raw materials sourced from both natural sites and cultivated ones. The proposed analytical methods for the determination of these compounds in plant material are fast and reliable and can be performed on every standard HPLC system.

## Figures and Tables

**Table 1:** Validation parameters of the HPLC-DAD analysis (*n* = 6).

Compound	Purity (%)	Precision intraday (CV, %)	Calibration equation	Linearity (*r*^2^)	Linear range (mg/mL)	LOD (*µ*g/L)	LOQ (*µ*g/L)
/+/-Catechin	98,3	0.80	*y* = 8216.4*x* − 6069.3	0.9995	0.492–49.150	11.07	36.10
Luteolin 8-C-glucoside (orientin)	99,4	1.12	*y* = 2407.3*x* − 2358.1	0.9998	0.497–49.700	36.84	122.83
Quercetin 3-O-rutinoside (rutoside)	91,4	1.61	*y* = 1434.0*x* − 5093.0	0.9999	0.907–90.669	74.60	248.80
Quercetin 3-O-galactoside (hyperoside)	95,0	1.48	*y* = 3435.5*x* − 6882.2	0.9999	0.384–38.400	35.16	117.20
Isorhamnetin-3-O-rutinoside	95,1	1.09	*y* = 2096.1*x* − 904.8	0.9998	0.380–38.000	29.31	97.69
Isorhamnetin-3-O-glucoside	95,3	1.02	*y* = 1940.0*x* − 897.4	0.9998	0.380–38.000	33.90	113.00
Kaempferol 3-O-glucoside (astragalin)	99,4	1.68	*y* = 2104.5*x* − 2426.3	0.9999	0.410–81.906	33.00	109.90
3-O-Caffeoylquinic acid (chlorogenic acid)	98,64	1.24	*y* = 6517.4*x* − 12016.6	0.9997	0.395–39.456	20.97	69.90

Primverin	95,0	1.18	*y* = 12488.0*x* − 3594.7	0.9999	0.407–40.660	7.95	26.52
Primulaverin	95,2	0.96	*y* = 2785.4*x* − 5313.2	0.9999	0.395–39.520	38.39	127.96

**Table 2:** The content of identified phenolic compounds in *P. veris* and* P. elatior* flowers (mg/100 g DW).

Identified phenolic compounds	*P. veris*			*P. elatior*		
	Mean	±sd	CV (%)	Mean	±sd	CV (%)
(+)-Catechin	312.11	±90.91^*∗∗*^	29.13	171.75	±57.26	33.34
Orientin	204.23	±78.03 ns	38.21	149.13	±57.16	38.33
Rutoside	630.83	±199.15	31.57	1025.96	±237.21^*∗∗*^	23.12
Hyperoside	nd			265.30	±122.80	46.29
Isorhamnetin-3-O-rutinoside	740.24	±337.09	45.54	1142.75	±401.76^*∗∗*^	35.16
Isorhamnetin-3-O-glucoside	448.45	±143.39^*∗∗*^	31.97	124.73	±54.02	43.31
Astragalin	185.07	±38.79^*∗∗*^	20.96	117.27	±34.81	29.68
Chlorogenic acid	72.84	±22.81 ns	31.31	55.38	±21.04	37.99

Values are the mean ± standard deviation (*n* = 8); ^*∗∗*^*p* < 0.01, ns: insignificant difference, nd: not detected, and CV: coefficient of variation.

**Table 3:** The content of identified phenolic compounds in *P. veris* and* P. elatior* roots (mg/100 g DW).

Identified phenolic compounds	*P. veris*			*P. elatior*		
	Mean	±sd	CV (%)	Mean	±sd	CV (%)
Primverin	1183.32	±212.52^*∗∗*^	17.96	110.31	±26.03	23.60
Primulaverin	536.16	±168.32^*∗∗*^	31.39	74.40	±33.35	44.83

Values are the mean ± standard deviation (*n* = 8); ^*∗∗*^*p* < 0.01, CV: coefficient of variation.
